# Investigation of a Novel Noninvasive Risk Analytics Algorithm With Laboratory Central Venous Oxygen Saturation Measurements in Critically Ill Pediatric Patients

**DOI:** 10.1097/CCE.0000000000001204

**Published:** 2025-01-16

**Authors:** Sarah A. Teele, Avihu Z. Gazit, Craig Futterman, William G. La Cava, David S. Cooper, Steven M. Schwartz, Joshua W. Salvin

**Affiliations:** 1 Division of Cardiovascular Critical Care Medicine, Department of Cardiology, Boston Children’s Hospital, Boston, MA.; 2 Division of Pediatric Cardiac Critical Care Medicine, Department of Critical Care Medicine, Pittsburgh Children’s Hospital, Pittsburgh, PA.; 3 Division of Cardiac Critical Care Medicine, Department of Pediatrics, George Washington University, Children’s National Hospital, Washington, DC.; 4 Computational Health Informatics Program, Boston Children’s Hospital, Boston, MA.; 5 Department of Pediatrics, Boston Children’s Hospital, Boston, MA.; 6 Department of Pediatrics, University of Cincinnati College of Medicine, The Heart Institute, Cincinnati Children’s Hospital Medical Center, Cincinnati, OH.; 7 Division of Cardiac Critical Care Medicine, Departments of Critical Care Medicine and Pediatrics, The Hospital for Sick Children and The University of Toronto Temerty Faculty of Medicine, Toronto, ON, Canada.

**Keywords:** cardiac output, central venous oxygen saturation, critical care, monitoring, pediatrics, predictive analytics

## Abstract

**BACKGROUND:**

Accurate assessment of oxygen delivery relative to oxygen demand is crucial in the care of a critically ill patient. The central venous oxygen saturation (Svo_2_) enables an estimate of cardiac output yet obtaining these clinical data requires invasive procedures and repeated blood sampling. Interpretation remains subjective and vulnerable to error. Recognition of patient’s evolving clinical status as well as the impact of therapeutic interventions may be delayed.

**OBJECTIVE:**

The predictive analytics algorithm, inadequate delivery of oxygen (IDo_2_) index, was developed to noninvasively estimate the probability of a patient’s Svo_2_ to fall below a preselected threshold.

**DERIVATION COHORT:**

A retrospective multicenter cohort study was conducted using data temporally independent from the design and development phase of the IDo_2_ index.

**VALIDATION COHORT:**

A total of 20,424 Svo_2_ measurements from 3,018 critically ill neonates, infants, and children were retrospectively analyzed. Collected data included vital signs, ventilator data, laboratory data, and demographics.

**PREDICTION MODEL:**

The ability of the IDo_2_ index to predict Svo_2_ below a preselected threshold (30%, 40%, or 50%) was evaluated for discriminatory power, range utilization, and robustness.

**RESULTS:**

Area under the receiver operating characteristic curve (AUC) was calculated for each index threshold. Datasets with greater amounts of available data had larger AUC scores. This was observed across each configuration. For the majority of thresholds, Svo_2_ values were observed to be significantly lower as the IDo_2_ index increased.

**CONCLUSIONS:**

The IDo_2_ index may inform decision-making in pediatric cardiac critical care settings by providing a continuous, noninvasive assessment of oxygen delivery relative to oxygen demand in a specific patient. Leveraging predictive analytics to guide timely patient care, including support for escalation or de-escalation of treatments, may improve care delivery for patients and clinicians.

**KEY POINTS**

**Question:** Is a predictive analytics algorithm (inadequate delivery of oxygen [IDo_2_] index) able to noninvasively assess central venous oxygen saturation (Svo_2_) in a critically ill patient population?

**Findings:** The IDo_2_ index was found to correlate with a set threshold Svo_2_ across a large cohort of critically ill children.

**Meanings:** Leveraging predictive analytics to guide timely clinical assessment may improve care delivery for patients and clinicians.

## BACKGROUND

Inadequate delivery of oxygen (IDo_2_) during periods of critical illness may lead to tissue ischemia and end-organ dysfunction. Ensuring the balance between oxygen demand and oxygen delivery (Do_2_) remains a challenge in critical care medicine as it generally relies on clinicians to interpret multiple pieces of data (1–3). Distilling these data may be imprecise; for example, physicians’ clinical assessment of cardiac index has been demonstrated to have poor correlation with concurrent thermodilution measurements in pediatric intensive care patients (4).

The central venous oxygen saturation (Svo_2_) is a biomarker that enables an estimate of a patient’s oxygen extraction ratio and is therefore considered a reliable indicator of adequacy of Do_2_ (5). Svo_2_ measurement below 50% in a biventricular circulation, or 25–35% in a patient with single ventricle physiology, typically reflects oxygen extraction approaching a critical Do_2_ threshold beyond which oxygen uptake becomes delivery dependent (6, 7). Further decrease in Do_2_ or increase in oxygen demand could result in life threatening end organ anaerobic metabolism. Increased risk of tissue dysoxia has been demonstrated when Svo_2_ levels are below 40% (8). In critical care settings, Svo_2_ measurements are intermittently obtained through blood samples from an indwelling catheter positioned in a patient’s main pulmonary artery or the superior vena cava. Acquiring these clinical data requires invasive procedures and repeat blood sampling. Recognition of a patient’s deteriorating clinical status as well as the impact of therapeutic interventions using this technique is inherently delayed.

Early and accurate identification of at-risk patients is critical for effective delivery of medical care, especially in busy, high acuity, and high complexity environments. Predictive analytics informed by large volume high fidelity physiologic data offer opportunities to support clinical teams working in these settings (9, 10).

## OBJECTIVES

**Figure 1.**
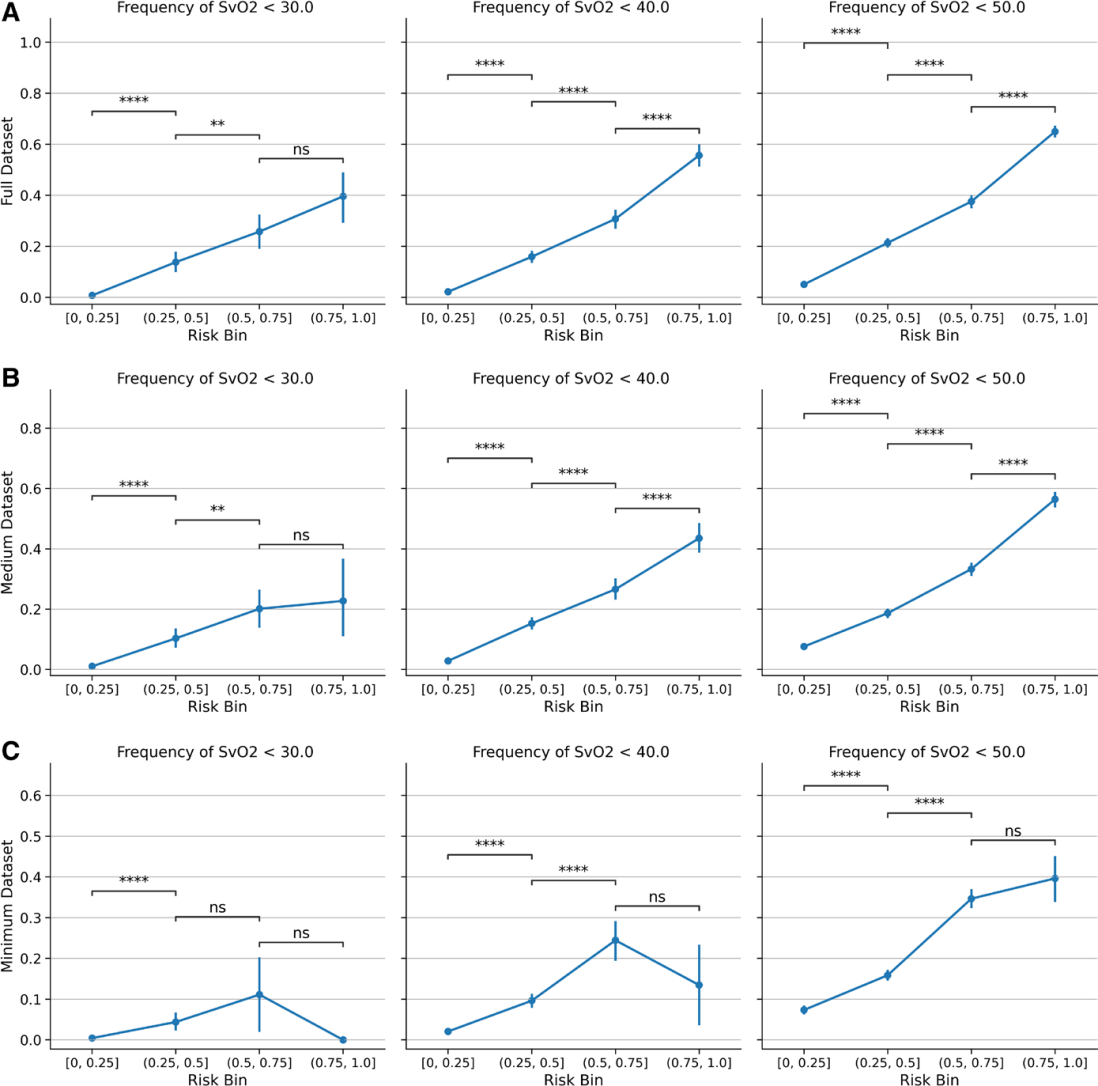
Values of central venous oxygen saturation (Svo_2_) observed within risk bins assigned by the inadequate delivery of oxygen (IDo_2_) score. Each *column* represents a different IDo_2_ index threshold (30%, 40%, and 50%). Each *row* corresponds to a different dataset: **A**, the full dataset; **B**, the medium dataset; and **C**, the minimum dataset. IDo_2_ scores are binned into quartiles, and the Svo_2_ values in adjacent bins are tested for significant differences using a Mann-Whitney-Wilcoxon test two-sided with Bonferroni correction. *p* value annotation legend: not significant (ns): 5e^–02^ < *p* ≤ 1; *1e^–02^ < *p* ≤ 5e^–02^; **1e^–03^ < *p* ≤ 1e^–02^; ***1e^–04^ < *p* ≤ 1e^–03^; ****p ≤ 1e^–04^.

A predictive analytics algorithm called the IDo_2_ index was developed by Etiometry (Boston, MA) to continuously calculate the likelihood of a critically ill pediatric patient’s Svo_2_ to fall below a specific threshold (30%, 40%, or 50%). Please see **Figures 1** and **2** in the **Supplemental Content** for details (http://links.lww.com/CCX/B456). An elevated IDo_2_ is associated with increased risk of cardiac arrest in neonates following cardiopulmonary bypass operations (11) and may provide an early warning for serious events in the general pediatric care patient population. This early warning could prompt a clinician’s focused attention and potentially inform the impact of therapeutic interventions. Given the important clinical implications for healthcare delivery in high acuity environments, the primary aim of this study was to assess the validity of IDo_2_ index as a tool to predict Svo_2_ thresholds of 30%, 40%, and 50%.

## MATERIALS AND METHODS

This was a retrospective multicenter cohort study conducted using data temporally independent from the design and development phase of the IDo_2_ index. Approval was obtained by Solutions Institutional Review Board (IRB) before the collection of data (IRB Registration Number: IORG0007116, November 23, 2022, Retrospective Validation of Updates to Etiometry Risk Analytics Technology). Procedures were followed in accordance with the ethical standards of the responsible committee on human experimentation and with the Helsinki Declaration of 1975. The patient population included critically ill postoperative neonates (0–28 d old), infants (29 d to 1 yr old), and children (> 1–12 yr old) with available Svo_2_ data who presented to ICUs at five tertiary care pediatric hospitals between February 7, 2016, and November 6, 2018 (**Table 1**). Additional data including vital signs, ventilator data, laboratory data, and demographics were acquired by T3 Data Aggregation & Visualization (Etiometry) software module and the institutional electronic medical record. Patient data were then de-identified, and the IDo_2_ index was retrospectively computed for Svo_2_ thresholds of 30%, 40%, and 50%. The average values of IDo_2_ for each threshold were computed for a period of 30 minutes leading to but excluding the time stamp of the measured Svo_2_. In a previous study, 10-, 20-, and 30-minute intervals were tested and found to be identical (11). The data from all patients were then collated for each discrete index threshold.

**TABLE 1.** Patients and Points Included in Validation Analysis

Institution	Date Range of Data Collection	Patients	Points	Points/Patient	Demographics (% Neonates % Infants % Children)
Site 1	September 1, 2016 to November 6, 2018	1,099	5,725	5.2	20 43 37
Site 2	February 7, 2016 to September 13, 2018	278	1,262	4.5	23 45 33
Site 3	August 25, 2016 to October 12, 2018	241	961	4.0	20 54 27
Site 4	March 27, 2017 to October 15, 2018	199	1,211	6.1	36 47 17
Site 5	September 1, 2016 to September 7, 2018	1,201	11,265	9.4	22 48 29
Total		3,018	20,424	6.8	22 46 31

IDo_2_ was categorized as positive or negative for each of the three detection thresholds (Svo_2_ values 30%, 40%, and 50%). A positive result indicated an Svo_2_ value less than the index threshold, whereas a negative result indicated a Svo_2_ value equal to the threshold setting or greater. To test the robustness of each index threshold and mimic ICU conditions where complete data may not be available, three datasets of varying completeness were generated. The complete data set contained all available data. The medium dataset contained all available data with the exception that Svo_2_ laboratory values were removed. Additional down sampling was done for the minimum dataset where pulse oximetry and arterial blood pressure were limited to one data point every 10 minutes and heart rate was limited to one data point every 60 seconds.

By using a Bayesian model, the IDo_2_ index bypasses the issue of missing data by calculating continuously maintained probability densities. When data are available, probability densities are updated to incorporate the new information. When data are not available, probability densities are produced by the relationships defined in the physiologic model, allowing the calculation to proceed.

Each IDo_2_ index threshold was evaluated for discriminatory power, range utilization, and robustness. To test the discriminatory power of each IDo_2_ index threshold, the area under the curve (AUC) of the receiver operating characteristic (ROC) curve was calculated for the full, medium, and minimum datasets. As the IDo_2_ index increased, it was expected that the observed Svo_2_ values would decrease. To test the range of each threshold, IDo_2_ values were binned into quartiles. The Svo_2_ values falling within adjacent IDo_2_ bins were compared using a two-sided Mann-Whitney-Wilcoxon test with Bonferroni correction. A monotonic decrease in Svo_2_ across the range would indicate that IDo_2_ scores correspond well to a higher risk of Svo_2_ being below the index threshold. The robustness for each IDo_2_ index threshold was evaluated by conducting the above analyses under three datasets of differing data availability.

All analyses were performed using custom scripts written in the Python programming language. Packages used include pandas (a toolkit for managing and processing time series data), SciPy (a toolkit that provides statistical analysis methods), and scikit-learn (a toolkit that provides machine learning and additional statistical methods for AUC analysis) (12–14).

## RESULTS

A total of 3,018 ICU patients contributed 20,424 Svo_2_ measurements for analysis (Table 1). The number of patients each study site contributed ranged from 199 to 1201, and the number of Svo_2_ samples per patient per site ranged from 4.0 to 9.4. Infants made up the largest proportion of the total population (46%). As the subjects increased in age, fewer instances of low Svo_2_ were observed (**Table 2**). For the 2–12-year-old age group, the available data points of Svo_2_ less than 30% were not sufficient for analysis.

**TABLE 2.** Patients and Data Points by Central Venous Oxygen Saturation and Age

Svo_2_ Data	Neonates	Infants	Children	All Ages
Total Svo_2_ data	6,559	9,660	4,205	20,424
Total Svo_2_ < 50%	1,267	1,985	350	3,602
Total Svo_2_ < 40%	444	597	127	1,168
Total Svo_2_ < 30%	114	135	Not applicable	249

Svo_2_ = central venous oxygen saturation.

The discriminatory power of the IDo_2_ index for predicting Svo_2_ below each specified threshold was assessed based on the ROC curves for each threshold and dataset. The 30% IDo_2_ index had the highest observed predictive ability across all datasets with AUCs (95% CI of 0.90 (0.88–0.92), 0.84 (0.81–0.87), and 0.81 (0.78–0.84) for the maximum, medium, and minimum datasets, respectively. The 40% IDo_2_ predictive threshold exhibited AUCs of 0.89 (0.88–0.90), 0.83 (0.82–0.84), and 0.78 (0.76–0.79) for the maximum, medium, and minimum datasets, respectively. The 50% IDo_2_ predictive threshold had observed AUCs of 0.86 (0.85–0.86), 0.78 (0.77–0.79), and 0.72 (0.71–0.73) for the maximum, medium, and minimum datasets, respectively. Datasets with greater amounts of available data had larger AUCs. This was observed across each configuration.

**Figure 1** details the observed values of Svo_2_ corresponding to binned IDo_2_ scores at each threshold for the study’s maximum, medium, and minimum datasets. As demonstrated, Svo_2_ values decrease as IDo_2_ score increases, with significant differences in Svo_2_ between all adjacent IDo_2_ score bins (*p* < 0.03). For the medium dataset, Svo_2_ values are significantly lower between all adjacent, increasing IDo_2_ quartiles (*p* < 0.01) except between the third- and fourth-quartile bins of IDo_2_ with a 30% threshold. In the minimum dataset, Svo_2_ values were significantly different between most IDo_2_ score bins, but for the three comparisons we observed no significant difference in Svo_2_ values, likely due in part to low sample sizes.

## DISCUSSION

### Predictability and Clinical Impact

The IDo_2_ index, a novel risk analytics algorithm, was found to correlate with a set threshold Svo_2_ across a large cohort of critically ill children. The predictive power of the algorithm is more robustly supported in the infant age group when compared with other age categories. Because the IDo_2_ index allows for continuous trend monitoring based on aggregate data points, the more inputs fed into the T3 software, the more accurately the algorithm may predict the likelihood of a low Svo_2_. Due to limited data in the IDo_2__30 dataset, it was not possible to demonstrate statistical significance for a monotonic increase across the IDo_2_ range.

### IDo_2_ Index and Decision-Making Strategies

By leveraging predictive analytics, the IDo_2_ index attempts to provide continuous assessment of Do_2_ relative to demand in an individual patient. Given the algorithm’s predictive and discriminatory power, the IDo_2_ index can inform decision-making in pediatric critical care settings. The IDo_2_ index may prompt early evaluation and action when the patient’s clinical state changes. This may result in more timely and targeted responses from clinicians, even in circumstances where invasive monitoring is not immediately available. Conversely, when the probability of such events is signaled to decrease, more rapid de-escalation of care may be possible, potentially reducing patient exposure to ICU morbidities and reducing unnecessary critical care costs (15). Clinical Decision Support Systems may choose to leverage the IDo_2_ index in their design, however, understanding how teams make clinical decisions in environmentally and culturally valid medical care contexts is critical if predictive analytics are to be used to their full potential. Diverse stakeholders should be involved when designing solutions that bridge technology with the human factor realities of healthcare delivery (16).

There are several limitations to the use of IDo_2_ in clinical practice. The IDo_2_ index performs better when more inputs are available; patients with limited physiologic data will have less reliable measures of IDo_2_. Although the IDo_2_ index was developed to predict the likelihood of Svo_2_ to fall under specific thresholds identified as clinically relevant in the above models, the threshold value may vary across heterogeneous patient populations. Like any biomarker, IDo_2_ should not be used in isolation to assess the clinical status of a patient. Furthermore, the predictive power of the IDo_2_ index could not be fully validated in specific subgroups of relatively rare patients due to insufficient data points.

This is a multi-institutional retrospective study using available Svo_2_ data. The specific location of the central venous line tip was not available and therefore there is a risk of inaccurate or contaminated sampling, a phenomenon more common in patients with complex congenital heart disease (CHD). In addition, there may have been mislabeled Svo_2_ samples; these potential errors can be harder to detect in patients with cyanotic CHD.

## CONCLUSIONS

The IDo_2_ index may offer a noninvasive, continuous near real-time assessment of Svo_2_ in critically ill patients. Future studies should be aimed at the association of IDo_2_ with clinical outcomes and the impact of active integration of predictive analytics into patient management.

## ACKNOWLEDGMENTS

We thank Michael McManus and Conor Holland for their support in article preparation.

## Supplementary Material
