# Supplementary material for: Investigation of a Novel Noninvasive Risk Analytics Algorithm With Laboratory Central Venous Oxygen Saturation Measurements in Critically Ill Pediatric Patients
Source: Crit Care Explor. 2025 Jan 16;7(1):e1204. doi: 10.1097/CCE.0000000000001204 (PMC11741213; doi:10.1097/CCE.0000000000001204)
Supplement: Supplementary file 1 [file cc9-7-e1204-s001.pdf]

IDO2 Manuscript Supplemental Content

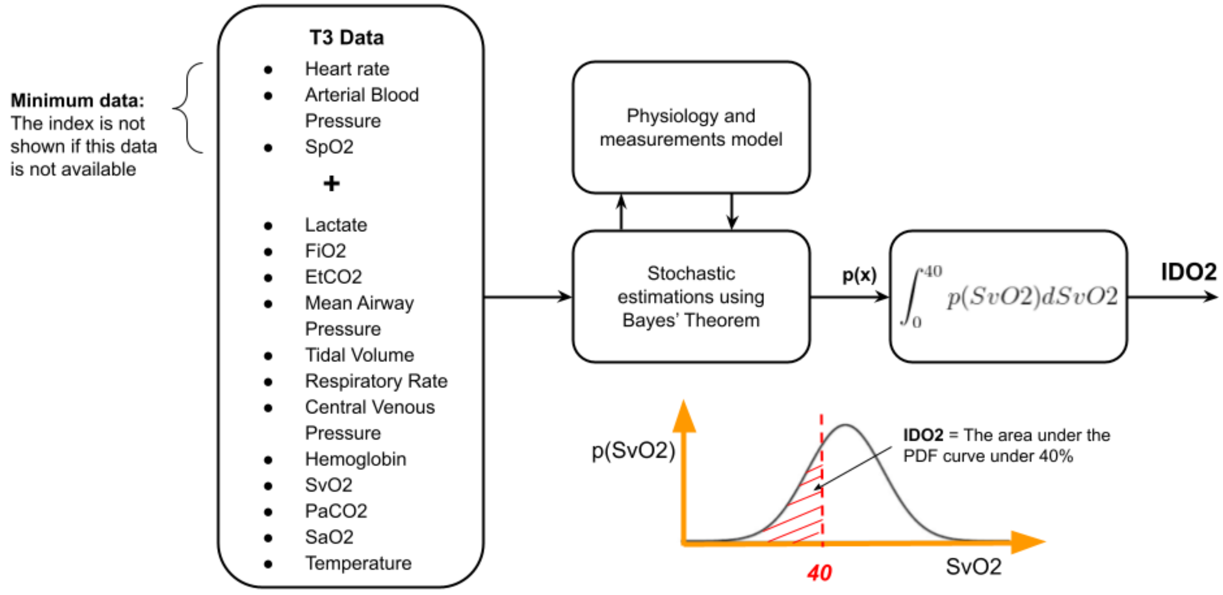

Figure 1: Data flow and computation details of the IDO2 Index

Index Calculation

The IDO2 Index has been designed to incorporate data acquired in near-real time by the T3 Software. As T3 continuously collects patient physiologic measures and laboratory test results, the algorithm uses Bayes' theorem to interpret the newly acquired data given its own previous assessment of the physiologic state of the patient. It then uses the interpreted data to update its assessment of the physiologic patient state. This patient state is represented as a set of patient physiologic variables, each of which is modeled with a probability distribution to account for patient-to-patient variation and measurement uncertainty. Physiologic variables representing the patient state are also related to one another using established relationships of human physiology. Finally, the physiologic variables are related to the physiologic measures and laboratory test

results collected by T3 using models of the measurement sensors that account for potential errors in the data. These elements make up the software physiology model.

Mixed venous oxygen saturation is one of the patient physiologic variables that the algorithm updates given new data. Specifically, the algorithm maintains an estimate of the probability density of SvO2. The IDO2 Index is calculated from this probability density by computing the cumulative probability of SvO2 between 0 and 30% (IDO2_30), 0 and 40% (IDO2_40), and 0 and 50% (IDO2_50). Figure 1 above shows the data flow and calculation details of the IDO2 Index (IDO2_40). It also depicts the minimum set of data required for the index to be calculated.

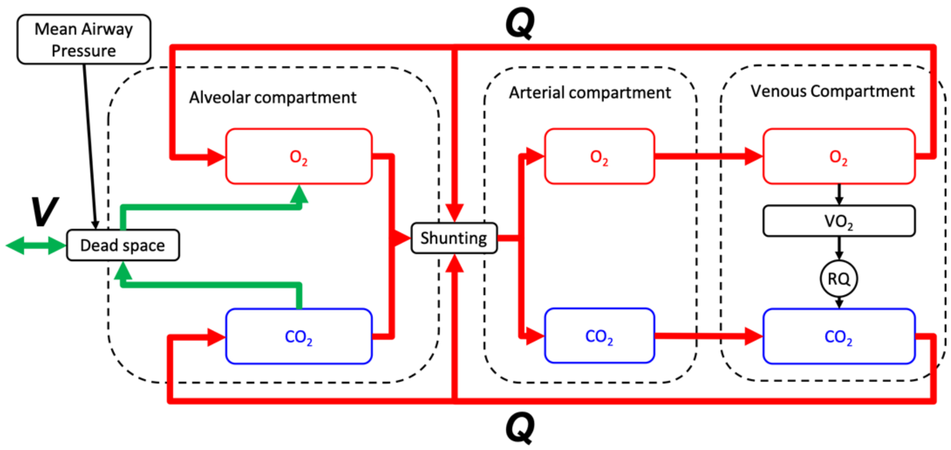

Figure 2: Components of Physiologic Model used for the IDO2 Index calculation

Physiologic Model

The algorithm behind the IDO2 Index employs a physiologic model that consists of two elements: 1) a dynamic model capturing the evolution of various physiologic variables in terms of nonlinear differential equations, and 2) a static model that relates some of the physiologic variables with the available measurements. The model captures gas exchange dynamics in terms

of two compounds (oxygen and carbon dioxide) circulating in three interconnected compartments: alveolar, arterial, and venous. The ventilation rate and cardiac output drive the exchange. V-Q mismatch is captured by the alveolar dead-space and the pulmonary shunting variables, which present idealized portions of the lung that are respectively ventilated but not perfused and perfused but not ventilated. Figure 2 depicts the components of this model.
